# Kerion-like lesions following an autoinoculation event in patient with chronic onychomycosis – Case report

**DOI:** 10.1016/j.mmcr.2024.100685

**Published:** 2024-11-23

**Authors:** Andrzej Kazimierz Jaworek, Przemysław Hałubiec, Paweł Marcin Krzyściak, Anna Wojas-Pelc, Jadwiga Wójkowska-Mach, Jacek Cezary Szepietowski

**Affiliations:** a Chair of Dermatology, Jagiellonian University Medical College, Botaniczna 3, 31-503, Cracow, Poland; b Doctoral School of Medical and Health Sciences, Jagiellonian University Medical College, Łazarza 16, 31-530, Cracow, Poland; c Chair of Microbiology, Jagiellonian University Medical College, Czysta 18, 31-121, Cracow, Poland; d Faculty of Medicine, Wroclaw University of Science and Technology, Grunwaldzki 11 sq., 51-377, Wroclaw, Poland

**Keywords:** Trichophyton rubrum, Tinea, Kerion, Two feet-one hand syndrome

## Abstract

We report a case of a 75-year-old male with suspected onychomycosis of the right hand and both feet who also developed kerion-like changes in the skin of his head and neck after recent inguinal hernia surgery. A mycological examination revealed the presence of *Trichophyton rubrum* in all affected sites. Treatment with oral terbinafine and topical isoconazole nitrate was started, resulting in a significant improvement in skin lesions. The case we present underscores the possibility of autoinoculation transmission of dermatophytes and the need for a careful evaluation of the coexistence of mycoses at different anatomical sites.

## 1 Introduction

Dermatophytes are a leading cause of fungal infections of the skin, nails, and hair, due to their ability to produce keratinases [1]. Diseases caused by dermatophytes are generally referred to as tinea (a Latin term for moth). The genera that preferentially target humans are known as anthropophilic dermatophytes [2]. In most cases, the skin’s immune system prevents their growth, hence, they require the presence of certain predisposing factors such as immunosuppression, skin trauma, or a sufficiently high temperature and humidity in the environment [3].

The presentation of dermatophyte infection depends on various factors, including geographic location, socioeconomic status, and the age of the host [4]. *Trichophyton rubrum* is the leading cause of most types of tinea, except tinea capitis (with the majority of cases due to *T. tonsurans* and *Microsporum canis*) and tinea barbae, which are mostly caused by zoophilic dermatophytes [5].

Onychomycosis is a fungal infection of the nail, also known as tinea unguium if caused by dermatophytes, and can affect the nail matrix, plate, or bed [6]. The disease has a higher prevalence among elderly patients, estimated to be approximately 50 % in subjects older than 70 years [7]. The most common type is distal and lateral subungual onychomycosis (DLSO), which leads to nail discoloration, hyperkeratosis, accumulation of subungual debris and subsequently onycholysis.

In this report, we present the case of an elderly patient with chronic tinea unguium of the right hand and feet caused by *T. rubrum* that extended to the beard and scalp area due to autoinoculation.

## 2 Case presentation

A patient is a 75-year-old farmer, who was urgently admitted to the dermatology hospital unit with suspected concomitant tinea capitis, tinea barbae and onychomycosis of the right hand and both feet (day 0). He had no history of previous dermatological treatment. However, he claimed that toenail abnormalities were present for about 30 years and that lesions within the fingernails of the right hand started about 20 years ago. Changes in head and neck skin occurred after a recent inguinal hernia surgery (at day −24).

**Fig. 1:**
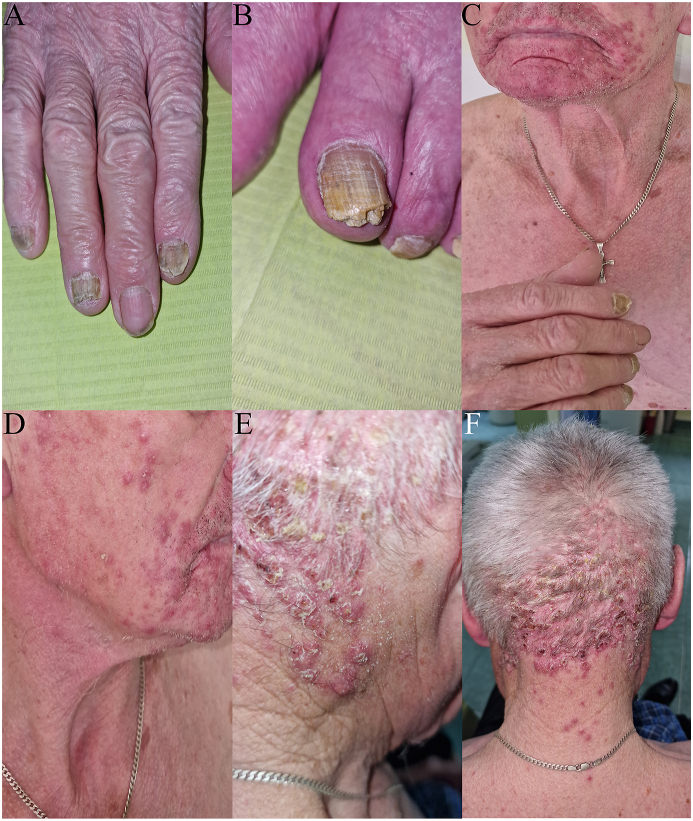
**Clinical images of nail and skin lesions in the patient presented. (A**–**B)** Nails present with yellow discoloration of the plates and transverse lines on their surface, subungual hyperkeratosis, and splitting and destruction of distal portions of the nail plates. **(C**–**D)** Confluent infiltrated erythematous lesions with pustules on the beard area. Singular diffuse erythematous papules covered with scale were present on the face skin. **(E**–**F)** Diffuse papules and pustules covered with yellow scales on the scalp area.

All nails of the feet and three nails of the right hand (II, IV and V) were involved. They presented yellow discoloration of the plate and transverse lines on its surface, subungual hyperkeratosis, splitting and destruction of the distal portions of the nail plates (Fig. 1A and B). With this clinical presentation, the two feet-one hand syndrome was suspected (the patient was right handed).

A confluent infiltrated erythematous lesions with pustules were found on the skin of the beard area (Fig. 1C and D). On the scalp, diffuse papules and pustules covered with yellow scales were also observed (Fig. 1E and F). These lesions were associated with persistent itch. There were no general symptoms or lymph node enlargement.

The material for mycological examination was obtained (on day 0) from scrapings of the affected nail plates and scales on the scalp, while the purulent material from the pustule in the beard area was sent for bacteriological and mycological evaluation.

Laboratory tests on day 0 showed mild normocytic anemia (HGB: 13.0 g/dL [N: 14.0–18.0], RBC: 4.1x10^6^/μL [N: 4.5–5.9], HCT: 38.7 % [N: 40.0–54.0]) and elevated C-reactive protein (27 mg/L [N: <5.00]). Other tests (electrolytes, glucose, liver, and kidney function tests) were normal. Virology tests for hepatitis B and C, as well as for human immunodeficiency virus, were negative.

Oral terbinafine (250 mg once a day) was started due to a positive result of the direct mycological examination (on day +1). Additionally, oral clindamycin (300 mg three times a day) was administered until the negative result of the bacteriological culture was received (day + 7). Topical 1 % isoconazole nitrate cream (combined with 0.1 % diflucortolone valerate until day +10) was applied to affected areas.

As the lesions resolved partially, the patient was discharged from the hospital (on day +4) with additional ambulatory management planned. Treatment tolerance was good with no complications.

Mycological examination of the feet and hand nail scrapes revealed the presence of *Trichophyton rubrum*. The same fungi species was cultured from purulent material from the beard area and the scales scrapped from the scalp.

On control visits (day +40 and + 120) the mild erythema persisted in the affected areas (without purulent exudate and scaling). Nails improved significantly and hyperkeratosis resolved. On control laboratory testing, HGB and CRP returned to normal. Topical isoconazole nitrate was maintained. The total time of treatment with terbinafine was 3 months.

## 3 Discussion

The diagnosis and treatment of tinea might sill pose a challenge for clinicians in more complicated cases [6]. Principles that guide diagnostic procedures must be strictly followed to avoid nondiagnostic samples or contamination, especially with respect to the location of the sample for culture (the margin of active skin lesions, whole nail clippings or scrapings, plucked hairs) [7]. A wide variety of auxiliary tools and techniques might be used to augment diagnostic accuracy. These include dermoscopy and Wood’s lamp in the clinic, while in the laboratory a polymerase chain reaction (PCR) or matrix-assisted laser desorption/ionization time of flight (MALDI-TOF) might be performed [3]. In case of diagnostic difficulties, a skin biopsy could be considered (usually it reveals parakeratosis, hyperkeratosis, spongiosis, neutrophils in the basal layer of the epidermis, and eosinophils in the dermis and ultimately fungal hyphae) [8]. Confirmation of diagnosis is critical in view of possible change of clinical presentation (referred to as tinea incognito) and increased resistance to further treatment if the initial approach was not optimized [9].

Treatment requires similar consideration and although some mild cases might respond to topical drugs alone, oral antifungals are required to achieve successful clearance of chronic skin and nail lesions (frequently in combination with topical agents) [10]. Currently, the allylamine drug terbinafine is considered a first choice in *T. rubrum* infections, although resistant strains have been described [11].

We present the case of an elderly patient who initially suffered from chronic tinea unguium and developed tinea capitis et barbae with kerion-like changes many years later after surgery. The likely explanation for the observed sequence is that dysregulation of immunity due to the burden associated with the operation allowed dermatophytes to grow in the beard and scalp area after accidental autoinoculation of chronically affected nails. Recent investigations show that male gender, elderly age of the patient, and concomitant tinea unguium are important factors that frequently coexist with the scalp and beard area infections [12]. Interestingly, some reports identified a female predominance among elderly patients with tinea capitis, indicating the role of impaired estrogen levels and following abnormalities in immune system function, as well as sebum production and composition [13].

Generally, *T. rubrum* is not the most typical fungal pathogen found in scalp lesions and kerion-like changes caused by *T. rubrum* are considered a unique clinical finding [12,[14], [15], [16]]. Other presentations, such as seborrheic dermatitis-like tinea capitis, were also found [17].

Tinea barbae is predominantly caused by *T. tonsurans* and *M. canis*; nonetheless, *T. rubrum* belongs to the most common etiologic factors among anthropophilic dermatophytes [18]. We managed to confirm the infection with *T. rubrum* in all involved areas.

The patient had a long history of onychomycosis that preceded the remaining skin lesions. Patients frequently do not seek professional advice on chronic nail abnormalities or do not continue the recommended treatment. Onychomycosis alone can cause a significant detriment to quality of life and cause (or exacerbate) other medical conditions such as thrombophlebitis or cellulitis [19]. It should be highlighted that the coexistence of chronic fungal infection in other anatomical regions (such as hairless skin, hands or feet) should be considered as an important clinical clue regarding the etiology of tinea capitis and tinea barbae.

The case we presented highlights the possibility of autoinoculation transmission of dermatophytes and the need for careful evaluation of coexistence of mycosis at different anatomical sites. Proper identification of the etiological factor of each of the lesions is important for a rational selection of treatment. Therapy should not be delayed due to the negative psychosocial and physiological consequences of onychomycosis [19]. Furthermore, patients who experienced such an extension of their initial disease should undergo a thorough evaluation to rule out the potential causes of immune system dysfunction (e.g. occult cancer, HIV infection, or malnutrition).

## CRediT authorship contribution statement

**Andrzej Kazimierz Jaworek:** Writing – review & editing, Resources, Methodology, Conceptualization. **Przemysław Hałubiec:** Writing – original draft, Visualization, Software, Resources, Methodology, Conceptualization. **Paweł Marcin Krzyściak:** Writing – review & editing, Validation. **Anna Wojas-Pelc:** Writing – review & editing, Supervision. **Jadwiga Wójkowska-Mach:** Writing – review & editing, Supervision. **Jacek Cezary Szepietowski:** Writing – review & editing, Supervision.

## Consent

We have obtained written and signed consent to publish the case report from the patient.

## Funding

This research did not receive any specific grant from funding agencies in the public, commercial, or not-for-profit sectors.

## Declaration of competing interest

There are none.
